# Topologically Optimized Copper Pre-Orienting Layer Enabled High-Quality GaN Micropyramid Epitaxy on Amorphous Glass

**DOI:** 10.3390/ma19143105

**Published:** 2026-07-20

**Authors:** Yaqing Ma, Junwei Cao, Huaze Zhu, Tong Jiang, Yuqiao Zheng

**Affiliations:** 1 Zhejiang University, Hangzhou 310027, China; mayaqing@westlake.edu.cn; 2 Advanced Solid-State Semiconductor Lab, School of Engineering, Westlake University, Hangzhou 310024, China; caojunwei@westlake.edu.cn (J.C.); zhuhuaze@westlake.edu.cn (H.Z.); jiangtong@westlake.edu.cn (T.J.); 3 College of Optical Science and Engineering, Zhejiang University, Hangzhou 310027, China

**Keywords:** gallium nitride, heteroepitaxy, selective-area epitaxy, amorphous substrates, metal pre-orienting layer

## Abstract

Heteroepitaxy of single-crystalline GaN on glass represents a promising approach for low-cost, large-area optoelectronics, yet the amorphous nature of glass inherently lacks the long-range atomic order required for crystalline templating. To address this limitation, we introduce a distinctive topologically optimized pre-orienting layer (TOPL) strategy based on Cu (111) thin films, enabling the growth of an AlN (0002) buffer layer and thus achieving high-quality GaN micropyramid array epitaxy on amorphous glass. First, by introducing a disconnected Cu thin film, the grain boundaries during annealing are effectively confined within the non-epitaxial region. This process to a large extent overcomes the polycrystallization of metal templates on amorphous glass. Then, experiments demonstrated that under multiply connected configurations, the Cu (111) surface not only preserves the grain-boundary-confining property but also exhibits notable surface smoothness. Among the evaluated symmetries, the sixfold symmetry structure achieves the optimum atomic-level flatness with a root-mean-square roughness of 0.395 nm. Building upon this TOPL architecture, c-axis-oriented GaN micropyramids were grown via selective area epitaxy. The glass-based GaN micropyramid demonstrates comparable crystalline quality (threading dislocation density from a single lamella: 5.46 × 10^8^ cm^−2^) to those grown on sapphire substrates, while exhibiting reduced stress and comparable cathodoluminescence full width at half maximum. Overall, the TOPL strategy presents a promising pathway toward low-cost GaN micropyramid epitaxy on glass substrates.

## 1. Introduction

As a representative third-generation semiconductor, gallium nitride (GaN) is of crucial importance for advancing next-generation optoelectronic devices and smart applications [1,2,3]. Conventional GaN epitaxy is highly dependent on rigid single-crystal substrates including sapphire [4,5,6,7,8], silicon [9,10], and silicon carbide [11,12,13,14,15,16]. These platforms facilitate stable III-V crystallization and effectively suppress rotational twinning, thereby enabling high-quality heteroepitaxial integration [17,18,19]. However, their inherent rigidity ultimately bottlenecks scalability and sustains high manufacturing costs for the GaN industry.

In contrast, glass-based optoelectronics offers a promising approach for achieving scalable and cost-effective devices [20,21]. Glass substrates exhibit exceptional optical transparency and excellent thermal and chemical stability, making them an attractive alternative to traditional single-crystal wafers. Nevertheless, the amorphous nature of glass fundamentally lacks the long-range atomic order required to template functional semiconductors, presenting a formidable challenge for the direct growth of high-quality, single-crystalline GaN films.

To bridge this structural gap, introducing a suitable pre-orienting layer on glass is an essential step. To date, two primary methodologies have been developed. The first involves transferring two-dimensional (2D) materials onto amorphous glass to serve as an epitaxial template [22,23,24,25,26,27,28], thereby enabling quasi-van der Waals epitaxy, or remote epitaxy is implemented [28]. The second relies on depositing thin-film pre-orienting layers, such as Titanium (Ti) [20,29,30], Ti/Tantalum nitride [31,32,33], Titanium nitride/Ti [34] and indium tin oxide [35], via electron-beam evaporation or sputtering, followed by selective area epitaxy (SAE) to guide GaN growth. Although nearly single-crystalline GaN and functional blue micro-LEDs have been successfully demonstrated on glass using these approaches, several critical limitations persist. For 2D material interlayers, their extreme thinness and low surface energy weaken the lattice-templating effect, typically resulting in a lower crystalline quality compared to conventional epitaxy on single-crystal substrates. More critically, under the high-temperature growth conditions required for GaN epitaxy (exceeding 1000 °C), 2D materials exemplified by graphene are susceptible to thermal degradation on AlN templates [21]. Meanwhile, and the precise control over the polarity of the resultant GaN films remains a significant technical hurdle [21]. This approach also suffers from high material costs, restricting its use to laboratory-scale research. On the other hand, conventional metal pre-orienting layers, such as Ti, typically suffer from a high density of in-plane grain boundaries [20]. Consequently, they necessitate SAE to confine GaN growth, yet the resulting GaN crystals still exhibit high defect densities that fall short of commercial standards.

Recently, bulk copper (Cu) has been explored for GaN epitaxy [36,37,38]. Meanwhile, Cu sheets have been reported to bypass the transfer step by directly growing a graphene pre-orienting layer for GaN epitaxy [39]. Our prior work demonstrated that bare Cu foil [40] or a Cu interlayer on conventional single-crystalline substrates enables direct GaN epitaxy [41]. Overall, Cu offers intrinsic advantages: first, the atomic arrangement of the Cu (111) surface exhibits a threefold symmetry that matches the lattice structure of III-nitrides, enabling it to guide epitaxial orientation [37]. Second, Cu films possess a much lower electrical resistivity (~17 nΩ·m) and a substantially higher thermal conductivity (~400 W/(m·K)) compared to Ti, which exhibits a resistivity of ~420 nΩ·m and a thermal conductivity of ~22 W/(m·K). These properties endow Cu with superior electrical conductivity and thermal management capabilities when integrated into device architectures as simultaneous contact electrodes. Finally, Cu is chemically stable, showing a minimal tendency to undergo adverse interfacial reactions with nitrides or substrates at elevated temperatures, thereby offering a wider processing window.

**Figure 1:**
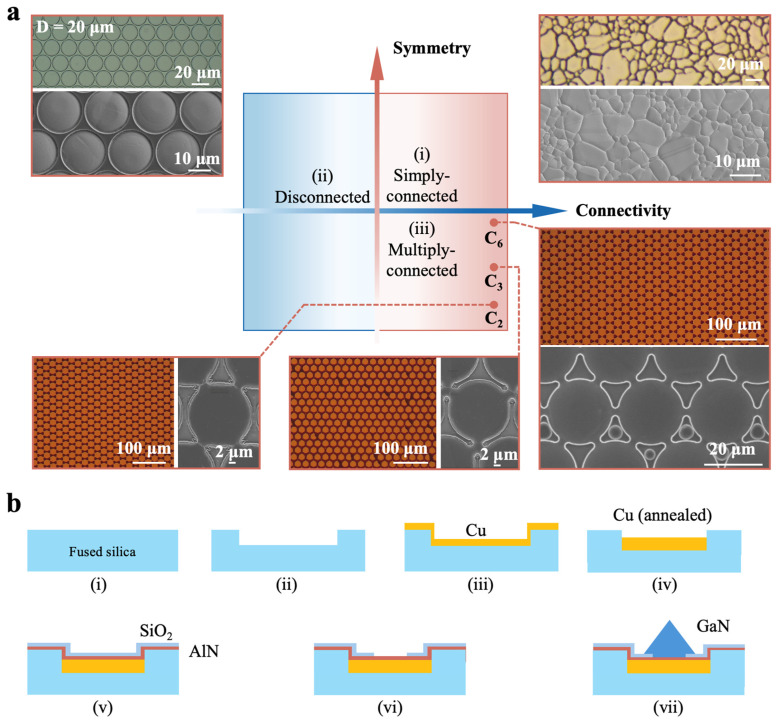
Schematic illustration of the TOPL-assisted GaN micropyramid epitaxy on amorphous glass. (**a**) Pre-orienting layer configurations investigated in this work. (**i**) Conventional simply connected thin film containing a high density of grain boundaries. (**ii**) Disconnected selective-area structure. (**iii**) Multiply connected selective-area structure under C_6_, C_3_ and C_2_ symmetries. (**b**) Process steps of GaN micropyramid epitaxy on amorphous glass. (**i**,**ii**) Amorphous glass substrate patterning. (**iii**) Cu deposition. (**iv**) Annealing to convert the polycrystalline Cu film into a textured Cu (111) pre-orienting layer and confine the layer within the pores. (**v**) AlN buffer layer and SiO_2_ mask construction. (**vi**) Epitaxial selective area construction. (**vii**) Two-step GaN micropyramid epitaxy.

This work introduces a topologically optimized pre-orienting layer (TOPL) strategy based on Cu (111) thin films to facilitate AlN (0002) buffer epitaxy, thereby achieving high-quality GaN micropyramid epitaxy on amorphous glass. By precisely tailoring the connectivity and symmetry, we achieved a Cu pre-orienting layer with atomic flatness and grain-boundary-free regions, which exhibits notable crystallinity and out-of-plane alignment. First, instead of adopting simply connected pre-orienting thin film (Figure 1(ai)), we designed a hexagonal selective-area configuration that effectively localizes Cu film grain boundaries at the edges of these areas under optimized structural parameters (Figure 1b), leaving the GaN growth regions virtually grain-boundary-free. Then, to resolve the surface roughness induced by surface discontinuities, we further established interconnection knots between the selective-area structures, creating multiply connected pre-orienting layers with sixfold (C_6_), threefold (C_3_) and twofold (C_2_) symmetries. Experimental observations indicate that under various symmetry conditions, the multiply connected configurations can maintain the grain-boundary-confinement property within the stress-concentrated non-epitaxial interconnection regions, while delivering an atomically flat surface. Leveraging the C_6_ configuration, which exhibits the optimum surface smoothness (root-mean-square, RMS: 0.395 nm), we achieved the epitaxial growth of high-quality, c-axis-oriented GaN micropyramids. The threading dislocation density (TDD) from a single micropyramid lamella reaches 5.46 × 10^8^ cm^−2^, meeting the qualification standards required for typical optoelectronic devices such as micro-LEDs [42,43]. Furthermore, compared to the GaN micropyramids grown on conventional sapphire substrates, those fabricated on the composite substrate exhibit a reduced internal residual stress, as evidenced by the lower Raman E_2_ (high) peak wavenumber. Meanwhile, the cathodoluminescence (CL) spectra from three independent samples display single, high-intensity near-band-edge (NBE) emission peaks in the range of 362.6 nm to 365.8 nm, with full-width at half-maximum (FWHM) values ranging from 16.9 nm to 17.5 nm. This demonstrates the high crystalline and optical qualities of the epitaxial material, as well as the satisfactory reproducibility of the TOPL-assisted epitaxial technique.

## 2. Materials and Methods

In this section, we introduce the materials, reagents, facilities setup and characterization conditions in this work.

Materials and reagents: The amorphous glass substrates (fused silica) and raw materials of Ga (7N), Al (6N) and Cu (6N) were purchased from ZhongNuo Advanced Material Technology Co., Ltd. (Beijing, China). The high softening temperature, strain point and low coefficient of thermal expansion of the fused silica ensures robust thermal shock resistance, preventing macroscopic deformation or viscous flow during the pre-orienting layer annealing and subsequent GaN growth processes. The N_2_ (99.999%), NH_3_ (99.999%) and HCl (99.999%) are purchased from Hangzhou BESTGAS Co, Ltd. (Hangzhou, China).

Template preparation: For both disconnected and multiply connected patterned Cu array structures, standard photolithography and reactive-ion etching were employed to open holes of various sizes on amorphous glass. Afterwards, 3–5 μm-thick Cu films were deposited onto the pre-patterned amorphous glass at 500 °C, followed by annealing at 1050 °C for 2 h in a flowing H_2_/Ar atmosphere. This process yielded Cu array structures with either disconnected or multiply connected holes, respectively.

Following the annealing steps, a 200 nm-thick aluminum nitride (AlN) buffer layer was deposited over the entire surface of Cu arrays using physical vapor deposition (Ar/N_2_ atmosphere, 150 W, 500 °C). Afterwards, a 200 nm-thick SiO_2_ layer was then deposited via plasma-enhanced chemical vapor deposition (PECVD, model: Samco PD-220NL, Samco, Kyoto, Japan). Standard photolithography and reactive-ion etching (RIE, model: RIE-230iP, Samco, Kyoto, Japan) were then used to open 3 µm-diameter patterned holes for subsequent GaN epitaxy.

Growth of GaN pyramid arrays: GaN pyramids were grown on the composite substrate via a two-step HVPE (home-built) process. Firstly, high-purity (7N) Ga metal was loaded in a pre-reaction chamber and reacted with HCl gas (gas flow: 40 sccm) at 900 °C to form GaCl precursor. The GaCl was then delivered by N_2_ carrier gas (gas flow: 1600 sccm) into the main reaction chamber, where it was reacted with NH_3_ (gas flow: 200 sccm) at 850 °C to grow a GaN buffer layer; the growth time was set for 2 min. Afterwards, the temperature of the reaction chamber was raised to 1000 °C. At this temperature, GaCl (HCl flow: 40 sccm, N_2_ carrier flow: 1600 sccm) reacted with NH_3_ (NH_3_ flow: 80 sccm, N_2_ carrier flow: 200 sccm) to form the GaN pyramid arrays.

Numerical simulations: A finite element method simulation based on COMSOL Multiphysics 6.1 was implemented, utilizing the coupled Heat Transfer in Solids and Solid Mechanics (Thermal Expansion) modules. The mesh size was configured as “Fine”. Both the copper and glass substrate, along with the selective area nodes, were designated as linear elastic materials. The dimensions are set according to the real dimensions of the structures. A fixed constraint was applied to the bottom boundary. Material properties were sourced from the built-in COMSOL material library, where “Copper [solid, residual resistivity ratio of 1000]” and “SiO_2_ (fused quartz) [solid, NIST SRM 739-Type I]” were selected for copper and glass, respectively. The simulation modeled the annealing process, which comprised heating from 25 °C to 1050 °C, holding at 1050 °C for 2 h, and cooling back to 25 °C.

SEM, TEM, XRD and AFM analysis: The surface morphology and microstructure of the samples, as well as electron backscatter diffraction (EBSD) mapping, were characterized using a Gemini 450 (ZEISS, Oberkochen, Germany) scanning electron microscope (SEM). The SEM images were taken under a working distance of 5.0 mm, an accelerating voltage of 3.00 kV and *I* probe of 200 pA. The EBSD images were taken under a working distance of 12.0 mm, an accelerating voltage of 20.00 kV and *I* probe of 10 nA. Transmission electron microscopy (TEM) samples of GaN pyramid arrays were prepared by focused ion beam (FIB) milling. Electron diffraction, bright/dark field imaging, and high-resolution TEM observations were performed using a Tecnai F20 transmission electron microscope at 200 kV (Thermal Fisher, Waltham, MA, USA). X-ray diffraction (XRD) rocking curve and in-plane-scan measurements of the GaN (0002), AlN (0002), and Cu (111) reflections were conducted using a Philips Xpertpro diffractometer with Cu-Kα radiation (Bruker, Karlsruhe, Germany). An AFM (Dimension ICON, Bruker, Billerica, MA, USA) was utilized to characterize the surface morphology of Cu films.

Grain boundary density evaluation: The grain boundary density is calculated as the ratio of the total grain boundary length (µm) to the total analyzed area (µm^2^).

CL and Raman measurement: CL measurements were conducted with a Hitachi scanning electron microscope (SU8600/Monnac Pro, Hitachi, Tokyo, Japan). The measurements were recorded under conditions of 5 kV and 10 μA, a scan range of 300–700 nm with a step size of 1 nm, and a dwell time of 2 s per point. The room-temperature Raman (WITec Alpha300RAS, WITec, Ulm, Germany) is carried out under an off-resonance light (405 nm continuous laser) under 10 mW power, with an injection spot size of approximately 1 μm^2^, facilitated by a ×50 objective lens with a numerical aperture of 0.55 (spectral resolution: 0.368 cm^−1^).

Summary among different sample groups investigated in this work: We involved several groups of samples in this work, and their key experimental conditions are summarized in Table 1.

## 3. Results

The process steps for TOPL-assisted GaN micropyramid epitaxy on amorphous glass are illustrated in Figure 1b, which mainly consist of (i,ii) amorphous glass substrates patterning; (iii) Cu deposition; (iv) high-temperature annealing to turn the polycrystalline Cu film into a textured Cu (111) pre-orienting layer and confine the Cu pre-orienting layer within the selective area holes. (v) AlN buffer layer and silicon dioxide (SiO_2_) mask deposition. (vi) SAE template construction; and (vii) two-step GaN epitaxy. We first investigate the effect of selective-area configurations on pre-orienting layer quality. This involves demonstrating the dependence of grain-boundary confinement efficiency on the diameters of the disconnected structures. Subsequently, by establishing multiply connected structures, we systematically evaluate the impact of different structural symmetries on the pre-orienting layer under identical knot widths and diameters, ultimately selecting the optimum configuration for subsequent GaN micropyramid array SAE.

### 3.1. Disconnected Cu Pre-Orienting Layer Annealing and Characterization

**Figure 2:**
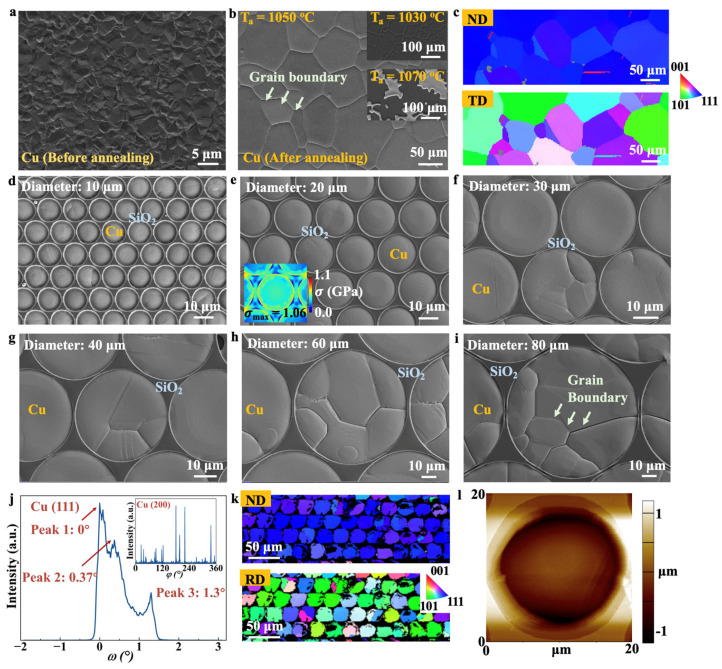
Characterization of the disconnected and simply connected Cu pre-orienting layer. (**a**) SEM image of the deposited Cu film on amorphous glass before high-temperature annealing. (**b**) SEM image of the Cu pre-orienting layer after high-temperature annealing at annealing temperatures of 1050 °C (S2), 1030 °C ((**inset-top**), S1) and 1070 °C ((**inset-bottom**), S3). (**c**) EBSD inverse pole figure mappings of deposited Cu pre-orienting layer after annealing, in the out-of-plane orientation (**top image**) mappings and in-plane orientation (**bottom image**). (**d**–**i**), SEM images of Cu pre-orienting layer retained within discrete selective area holes of varying diameters (10–80 μm) after high-temperature annealing. Inset in figure (**e**): Numerical simulations of stress distributions in Cu films within selective-area holes. (**j**) ω-scan of Cu (111) in selective-area arrays. Inset: φ-scan of Cu (200). (**k**) EBSD orientation maps of the Cu pre-orienting layer, displaying the out-of-plane orientation (normal direction, ND, (**top**)) and in-plane orientation (rolling direction, RD, (**bottom**)). (**l**) AFM image of Cu film within the selective area hole.

We demonstrate that although establishing size-optimized, disconnected selective-area holes can provide a certain degree of grain boundary confinement, it introduces a severe convex meniscus effect. First, we optimized the Cu annealing temperature (T_a_). Similar to the reported Ti pre-orienting layers [20,30], in a simply connected configuration (Figure 2a), the Cu film deposited on amorphous glass exhibits a highly uniform out-of-plane orientation after high-temperature annealing because Cu possesses the lowest surface energy along the (111) direction (Figure 2b). Notably, the annealing temperature is critical to the quality of the resulting Cu film. Previous studies have demonstrated that polycrystalline Cu films can transform into a single-crystalline state under an annealing condition of 1020 °C [44]. Herein, we discovered that further elevating the annealing temperature on the amorphous glass substrate can reduce the grain-boundary density to a certain extent. Specifically, the average grain size reaches approximately 50–100 μm (grain boundary density: 0.026 μm/μm^2^) at 1050 °C (Figure 2b, S2), whereas it ranges from approximately 20 to 80 μm (grain boundary density: 0.031 μm/μm^2^) at T_a_ = 1030 °C (Figure 2b: top inset, S1). However, a further temperature increment to 1070 °C induces severe volatilization of Cu atoms (Figure 2b: bottom inset, S3), as it approaches the melting point of Cu (1082 °C). Consequently, 1050 °C was selected as the optimum Cu annealing temperature in this study.

Overall, compared to Ti, Cu benefits from a lower melting point, which facilitates a reduction in grain boundary density through high-temperature annealing. However, because the annealing process is accompanied by the volatilization of Cu atoms, the annealing duration must be confined within 4 h, leaving randomly distributed twin boundaries with sizes ranging from 50 to 100 μm on the surface (Figure 2b,c), where the distinct color domains in the inverse pole figure (IPF) maps correspond to different crystallographic orientations (Figure 2c). To address this issue, we fabricated hexagonal selective-area hole arrays with varying diameters from 10 to 80 μm on glass substrates (Figure 2d–i, S6–S11) and apply the identical process for Cu film sputtering and annealing. It was observed that the originally continuous Cu film was completely confined within the selective holes after high-temperature annealing. When the diameter of the selective holes is relatively large (exceeding 60 μm), the grain boundaries still exhibit a random distribution. As the hole diameter decreases to 20 μm, the grain boundary density is significantly reduced (Figure 2d,e), confirming that the closed selective-area configuration is effective in collecting and confining grain boundaries. Meanwhile, despite the presence of a certain degree of twinning (evidenced by the peaks at 0°, 0.37°, and 1.3° in Figure 2j), the out-of-plane orientation remains largely uniform (Figure 2k-top), displaying distinct in-plane orientations similar to those observed in continuous Cu films (the inset of Figure 2j,k-bottom).

However, the disconnected selective-area structures induced severe agglomeration of the Cu film (Figure 2l). This phenomenon is driven by the thermodynamic minimization of the total surface and interfacial free energies during high-temperature annealing [45,46]. In these isolated micro-domains, the high surface-to-volume ratio drives the migration of Cu atoms toward the center of each hole, ultimately forming a highly curved, quasi-spherical surface morphology (Figure 2l). Such a non-uniform and non-planar surface poses a formidable obstacle to subsequent GaN microstructure epitaxy.

### 3.2. Multiply Connected Cu Pre-Orienting Layer Annealing and Characterization

**Figure 3:**
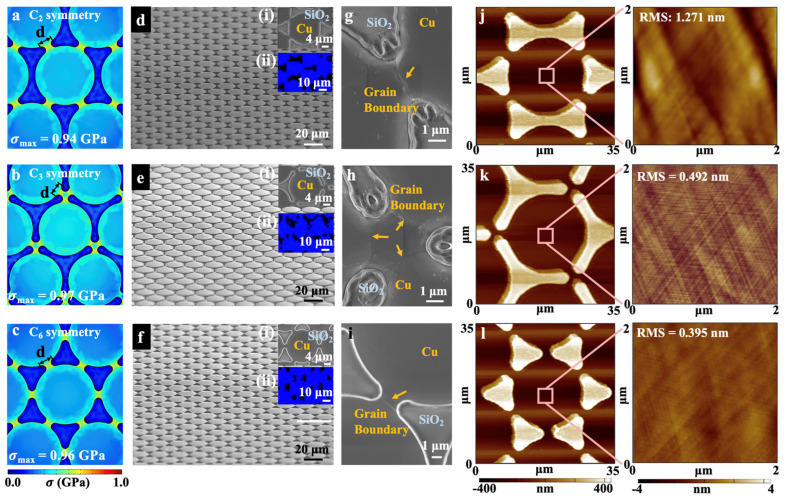
Characterization of the multiply connected Cu pre-orienting layers. (**a**–**c**) Numerical simulations of stress distributions of the multiply connected Cu pre-orienting layers under (**a**) C_2_, (**b**) C_3_ and (**c**) C_6_ symmetry configurations. (**d**–**f**) SEM images of the multiply connected Cu pre-orienting layers under (**d**) C_2_ (S12), (**e**) C_3_ (S13) and (**f**) C_6_ (S14) symmetry configurations. Insets: (**i**) magnified views of the selective area holes. (**ii**) EBSD orientation maps of the Cu pre-orienting layer. (**g**–**i**) SEM images of the confined grain boundaries (yellow arrows) of the multiply connected Cu pre-orienting layers under (**g**) C_2_, (**h**) C_3_ and (**i**) C_6_ symmetry configurations. (**j**–**l**) (**Left**): AFM images of the multiply connected Cu pre-orienting layers under (**j**) C_2_, (**k**) C_3_ and (**l**) C_6_ symmetry configurations. (**Right**): corresponding RMS roughness of the Cu pre-orienting layers.

To resolve the issue of convex meniscus surface, we designed a multiply connected configuration by establishing interconnecting knots between the closed selective-area holes, thereby achieving an atomically flat Cu pre-orienting layer surface while concurrently regulating grain boundaries (Figure 3a–c). Based on 20-μm selective holes under the same knot width (d) of 5 μm, we introduce multiply connected structures with C_2_ (Figure 3a), C_3_ (Figure 3b), and C_6_ (Figure 3c) symmetries based on the geometric characteristics of the hexagonal lattice. Compared with the disconnected hexagonal arrays where stress is relatively uniformly distributed along the edge (Figure 2e: inset), the multiply connected structures generate localized stress hotspots at the interconnecting knots (Figure 3a–c). Meanwhile, our experimental observations reveal that under all three symmetrical configurations, the annealed Cu films achieve high surface flatness (Figure 3d–f), as well as c-axis orientations along (111) (Figure 3d–f: inset-(ii)). This improvement is attributed to the interconnecting knots disrupting the inward material contraction dynamics driven by the minimization of surface free energy within the isolated micro-domains, thereby effectively suppressing the solid-state dewetting and surface agglomeration induced by localized capillary instabilities [47]. During this process, the efficacy of the selective holes in collecting and confining the Cu grain boundaries during annealing is preserved, with the internal grain boundaries being confined and strongly pinned within the knots (Figure 3g–i). Furthermore, we characterized the surface smoothness within a 2 μm × 2 μm area comparable for subsequent GaN SAE. Atomic force microscopy (AFM) results indicate that increasing the order of the geometric symmetry can further mitigate surface fluctuations and enhance surface flatness (Figure 3j–l). Consequently, for subsequent GaN heteroepitaxy applications, the C_6_ multiply connected selective-area structure, which exhibits the optimum surface flatness with an RMS roughness of 0.395 nm, was selected as the epitaxial template.

### 3.3. GaN Epitaxy on Amorphous Glass Based on Multiply Connected Cu Pre-Orienting Layer

**Figure 4:**
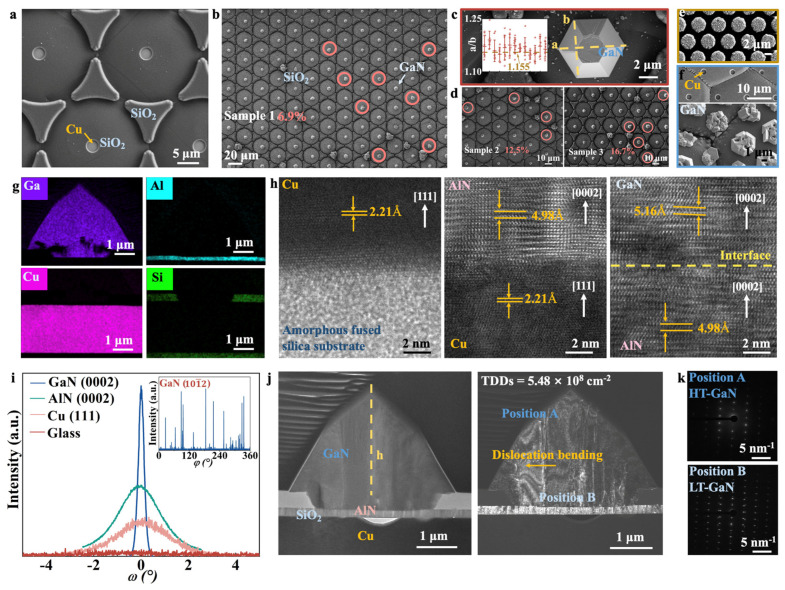
Characterization of the TOPL-assisted epitaxial GaN micropyramids on different substrates. (**a**) SEM image of the SiO_2_ selective-area arrays. (**b**) SEM image of the epitaxial GaN micropyramid array grown on AlN/C_6_ symmetric multiply connected Cu/amorphous glass heterostructure (S14), with twinned micropyramids marked and their percentage noted. (**c**) Zoom-in SEM image of the GaN micropyramid grows on AlN/C_6_ symmetric multiply connected Cu/amorphous glass heterostructure. Inset: distribution of the across-corner-to-across-flats ratio of the GaN micropyramids. (**d**) SEM images of two S14 samples, with twinned micropyramids marked and their percentage noted. (**e**) SEM image of the SAE GaN grains grown on a bare fused silica substrate (S5). (**f**) SEM image of the SiO_2_ selective-area arrays on simply connected Cu pre-orienting layer (**top**), and the GaN grains grown based on the AlN/simply connected Cu/amorphous glass heterostructure (S4). (**g**) Elemental distributions of one GaN micropyramid. (**h**) High-resolution TEM images of the Cu/amorphous fused silica interface (**left**), Cu/AlN interface (**middle**) and GaN/AlN interface (**right**). (**i**) XRD *ω*-scan (normalized) of the GaN/AlN/C_6_ symmetric multiply connected Cu/amorphous glass heterostructure. Inset: *φ*-scan (normalized) of the GaN grown on AlN/C_6_ symmetric multiply connected Cu/amorphous glass heterostructure. (**j**) Cross-sectional high-angle annular dark-field scanning images (**left**) and corresponding dark-field image (**right**) of GaN/AlN/Cu on amorphous glass heterostructure. (**k**) SAED patterns from positions A (**top**) and B (**bottom**) in (**j**). LT: low-temperature; HT: high-temperature.

The multiply connected selective-area configuration, with optimized grain boundary regulation and surface planarization, provides effective template support for c-axis-orientated GaN micropyramids growth. We first sputtered a 200 nm AlN seed layer onto the C_6_ symmetry Cu pre-orienting layer and fabricated a SiO_2_ mask containing micro-hole arrays on the amorphous AlN/Cu/glass heterostructure (Figure 4a, diameter: 3 μm). With growth initiated from these micro-holes, a two-step SAE process was implemented to yield GaN micropyramid arrays on amorphous glass (Figure 4b). The as-grown micropyramids exhibit a highly regular, geometrically well-defined hexagonal pyramidal morphology with sharp crystalline facets (Figure 4c), featuring an across-corner (a) to across-flat (b) ratio of 1.158, which is close to the theoretical value (1.155). Such morphological uniformity is preserved over a large area, with the ratio values distributed between 1.116 and 1.247 (Figure 4b, inset, where each bar group corresponds to a column of GaN grains). Notably, only about 12% of the twinned GaN grains are observed, which is determined by evaluating the proportion of twinned GaN grains relative to the total number of grains within the characterized region across three independent batches of S14 samples (Figure 4d; left: samples 2, right: samples 3). In contrast, patterning openings on a simply connected Cu film (Figure 4e, top) results in a pronounced polycrystalline configuration for the epitaxial GaN grains due to the randomly distributed grain boundaries (Figure 4f, bottom). In future investigations, the uniformity of the gas flow distribution can be enhanced through reactor structural optimization and gas ratio tuning, thereby further improving the GaN morphological uniformity and reducing the number of twinned grains.

The GaN micropyramids grown via the TOPL-assisted epitaxial technology exhibit distinct heterostructures and sharp elemental distributions, with no significant interdiffusion observed between the constituent layers (Figure 4g). Meanwhile, well-defined interfaces are observed, as illustrated by the high-resolution TEM at Cu/amorphous glass (Figure 4h-left), AlN/Cu (Figure 4h-middle), and GaN/AlN (Figure 4h-right) interfaces. Specifically, the XRD ω-rocking curve demonstrates a strong out-of-plane epitaxial relationship within the GaN/AlN/C_6_ symmetric multiply connected Cu/glass heterostructure (Figure 4i), although the in-plane rotation angles among different GaN micropyramids are inconsistent according to the XRD φ-rocking curve (the inset of Figure 4i).

TEM and corresponding dark-field imaging of an individual GaN micropyramid lamella reveal a low TDD of 5.46 × 10^8^ cm^−2^ (Figure 4j), matching the crystalline quality achieved via conventional heteroepitaxy on single-crystal substrates [48]. It can be observed from the TEM image that at the low-temperature buffer/high-temperature GaN interface, some dislocation lines exhibit a distinct change in direction. Specifically, the sharp and well-defined diffraction spots at the outer high-temperature epilayer (position A, Figure 4k) confirm favorable lattice integrity and high crystallinity, revealing enhanced crystal quality compared to the low-temperature buffer epilayer at the interface (position B, Figure 4k). On the other hand, due to a certain degree of gas flow non-uniformity, the height (*h*)-to-a ratio (0.390) of the micropyramids significantly deviates from the standard value for GaN grains (0.832), further underscoring the necessity of optimizing gas flow distribution. Overall, our findings demonstrate the important role of the low-temperature buffer layer in the two-step epitaxy process for mitigating stress, reducing threading dislocations, and ultimately enhancing the overall quality of the epitaxial layer.

**Figure 5:**
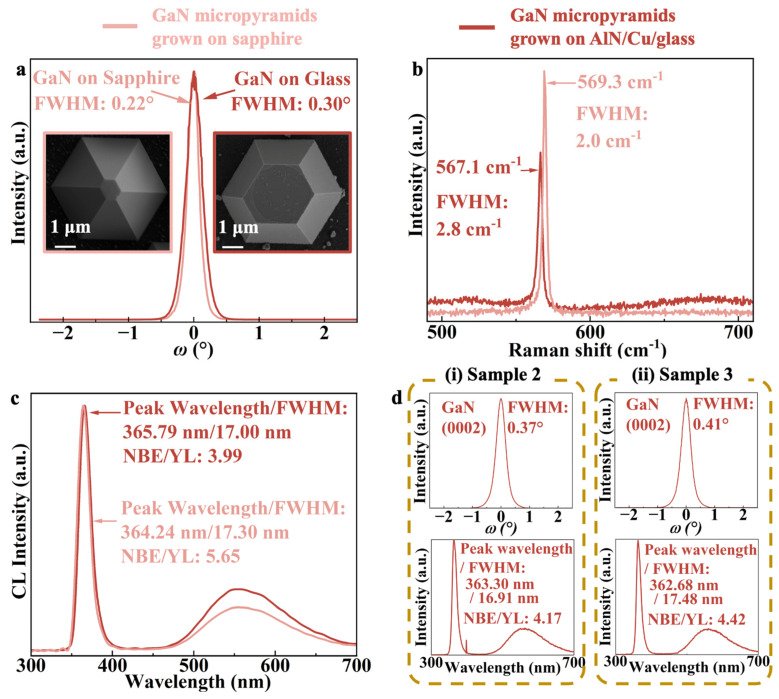
Comparison of the epitaxial GaN micropyramids on amorphous glass (S14) and sapphire (S15). (**a**) ω-scan of GaN (0002) grown on sapphire and AlN/C_6_ symmetric multiply connected Cu/amorphous glass heterostructure. Inset: GaN micropyramid grown on sapphire (**left**) and Cu-glass substrates (**right**). (**b**) Raman spectra of GaN grown on sapphire and Cu-glass substrates. (**c**) CL spectra of GaN grown on sapphire and Cu-glass substrates. (**d**) ω-scan and CL spectra of two GaN (0002)/AlN/C_6_ symmetric multiply connected Cu/amorphous glass heterostructure samples ((**i**): sample 2; (**ii**): sample 3).

Further crystalline quality characterization by XRD indicates that the FWHM of the (0002) ω-scan rocking curve for the glass-based GaN micropyramids is comparable to that of the sapphire-based counterpart (S15) under the same SAE mask openings (a diameter of 3 μm and a spacing of 22 μm), yielding values of 0.30° and 0.22°, respectively (Figure 5a). In contrast to the sapphire-based GaN grains that display pronounced compressive stress as evidenced by the Raman E_2_ (high) peak positioned at 569.3 cm^−1^ (FWHM: 2.0 cm^−1^, biaxial stress: −0.42 GPa), the glass-based GaN micropyramids exhibit a mitigated, low tensile stress state, with the peak located at 567.1 cm^−1^ (Figure 5b, FWHM: 2.8 cm^−1^, biaxial stress: +0.093 GPa), closely approaching the stress-free value (567.5 cm^−1^). This can be attributed to two main factors. On one hand, the amorphous glass possesses a lower coefficient of thermal expansion compared to GaN. On the other hand, our previous work has demonstrated that utilizing Cu thin films for GaN epilayer growth on single-crystalline substrates (sapphire, Si and SiC) leverages a unique self-relaxation mechanism, which is brought by the compliant metallic nature [41], thereby significantly enhancing the crystalline quality of the epitaxial film. In this work, we further extend this stress relaxation effect to amorphous substrate systems, thereby demonstrating the advantages of utilizing Cu as a pre-orienting layer. Meanwhile, CL spectra demonstrate that the FWHM of the NBE emission peak for the glass-based GaN grains is comparable to that of the epilayer grown on sapphire, although its NBE-to-yellow-luminescence (YL) intensity ratio is slightly higher than that of the sapphire-based epitaxial structure (Figure 5c). This YL emission is primarily ascribed to native deep-level defects and vacancy-impurity complexes acting as deep acceptors, which are commonly introduced during the heteroepitaxy of unintentionally doped GaN [20]. Meanwhile, two other independent batches of grown samples (S14, corresponding to the samples in Figure 4d) also exhibit satisfactory material quality, with their FWHM values of the GaN (0002) ω-scan rocking curve being 0.37° and 0.41°, respectively. Moreover, these samples possess similar CL NBE emission peaks and NBE-to-YL ratios (Figure 5d), demonstrating the favorable reproducibility of the proposed TOPL-assisted epitaxial technology.

Overall, our results validated that a C_6_ symmetry multiply connected Cu interlayer successfully overcomes the crystalline limitations of amorphous substrates, presenting a promising materials platform for further exploration toward GaN-based optoelectronic applications on glass.

## 4. Conclusions and Discussion

In summary, to address the scalability constraints imposed by the dependence of GaN epitaxy on single-crystal substrate, as well as the degraded crystalline quality caused by grain boundaries within conventional pre-orienting layers on amorphous substrates, this work proposes a TOPL-assisted epitaxial technology based on a Cu interlayer. By fabricating a C_6_ symmetric multiply connected Cu pre-orienting layer, we regulate the mass transport pathways, thereby effectively suppressing solid-state dewetting and surface agglomeration induced by discrete selective area configuration. This approach yields an atomically flat Cu template surface (RMS: 0.395 nm) while confining the grain boundaries within the interconnecting knots. Utilizing this optimized template, an AlN (0002) buffer layer is grown, upon which a two-step SAE produces highly regular, c-axis-orientated GaN micropyramid arrays on amorphous glass. TEM analyses of a single GaN micropyramid grain confirm excellent lattice integrity and a low TDD, matching the crystal quality achieved via conventional heteroepitaxy on single-crystal substrates. Ultimately, this topological engineering of the Cu pre-orienting layer presents a promising route toward GaN micropyramid growth on glass templates, paving the way for functional device development.

For future optoelectronic device applications, several critical factors are worthy of meticulous attention. On one hand, although EDS confirms no obvious interfacial diffusion, potential defect-assisted Cu migration via the kick-out mechanism will be mitigated in device integration by further optimizing the AlN buffer density or incorporating ultra-thin diffusion barriers. Additionally, optimizing the Cu interlayer thickness remains a critical focus, with particular emphasis on investigating the dependence of grain-boundary confinement and planarization effects at lower thicknesses to facilitate device integration. On the other hand, the effects of other geometric parameters (e.g., diameter, knot width, or fill factor) of the multiply connected regions under various symmetries on the quality of the Cu pre-orienting layer remain unexplored, which is worthy of further detailed investigation. More importantly, a theoretical model describing the impacts of annealing stress distribution on grain-boundary migration across different morphologies has not yet been established; developing such models will provide crucial guidance for GaN epitaxy on amorphous substrates and benefit the scalability and cost reduction in GaN-based devices.

## Figures and Tables

**Table 1:** Experiment conditions of sample groups investigated in this work.

Sample Group (Group Name)	Cu Depositing Power/Temperature/Time	Annealing Temperature/Time	AlN Depositing Power/Temperature/Time	LT-GaN Epitaxial Temperature/Time	HT-GaN Epitaxial Temperature/Time
Simply connected (S1)	120 W/500 °C/1.5 h	1030 °C/2 h	-	-	-
Simply connected (S2)		1050 °C/2 h	-	-	-
Simply connected (S3)		1070 °C/2 h	-	-	-
Simply connected (S4)		1050 °C/2 h	150 W/500 °C/40 min	850 °C/2 min	1000 °C/3 min
Simply connected (S5)	-	-			
Disconnected (S6–S11)	120 W/500 °C/1.5 h	1050 °C/2 h	-	-	-
Multiply connected C_2_ (S12) and C_3_ (S13)			-	-	-
Multiply connected C_6_ (S14)			150 W/500 °C/40 min	850 °C/2 min	1000 °C/3 min
Bare sapphire (S15)	-	-			

LT: low-temperature; HT: high-temperature.

## Data Availability

The original contributions presented in this study are included in the article. Further inquiries can be directed to the corresponding author.
